# Respiratory Involvement During the Course of Hypocomplementemic Urticarial Vasculitis: A Case Report

**DOI:** 10.7759/cureus.89813

**Published:** 2025-08-11

**Authors:** Leroy Dorian, Rudi Peché, Julien Guiot, Sebastien Van Laethem

**Affiliations:** 1 Pulmonary Medicine, Université Libre de Bruxelles, Brussels, BEL; 2 Pulmonology, Humani Charleroi - Université Libre de Bruxelles, Charleroi, BEL; 3 Pulmonology, Centre Hospitalier Universitaire (CHU) Liège - University of Liège (ULG), Liège, BEL

**Keywords:** bronchiolitis, hypocomplementemic, mac duffie syndrome, obstructive disorder, vasculitis

## Abstract

Hypocomplementemic urticarial vasculitis syndrome is a rare small-vessel vasculitis characterized by the combination of vasculitis and chronic urticaria. The hypocomplementemic form is distinguished by a more severe clinical presentation than the normocomplementemic form, with prognosis influenced by respiratory involvement. Due to its rarity and the similarities in presentation to systemic lupus erythematosus (SLE), diagnosis may be delayed and treatment may be inappropriate. We report the case of a young non-smoking female patient with a history of SLE refractory to standard treatments, whose progression was marked by severe and irreversible obstructive ventilatory disorder. This case aims to highlight the potential for severe and irreversible obstructive disorders that may occur in this condition in young non-smoking patients.

## Introduction

Hypocomplementemic urticarial vasculitis syndrome (HUVS), also known as McDuffie syndrome, is a rare autoimmune small-vessel vasculitis characterized by recurrent urticarial lesions, low serum complement levels, and potential multisystem involvement. While normocomplementemic urticarial vasculitis is typically confined to the skin and considered benign, HUVS often presents with systemic manifestations, including arthritis, ocular inflammation, glomerulonephritis, and pulmonary complications. Among these, respiratory involvement, though infrequent, is a major determinant of prognosis and can include obstructive airway disease, alveolitis, or emphysematous changes. Despite its severity, the pulmonary component remains poorly documented, largely due to the rarity of HUVS. Emerging reports suggest a possible correlation between smoking history and the severity of lung involvement, particularly in middle-aged adults [1]. In this report, we present a clinical case of HUVS in a young non-smoking female patient with significant pulmonary manifestations, aiming to highlight the radiologic, functional, and clinical features of respiratory involvement in this underrecognized syndrome.

## Case presentation

A 30-year-old non-smoking female presented to the pulmonology department with exertional dyspnea that had been progressively worsening over several months. Her medical history included fluctuating non-allergic conjunctivitis and systemic lupus erythematosus (SLE), diagnosed based on a high titer of antinuclear antibodies (1:2560), positivity for anti-double-stranded DNA, SSA, and SSB antibodies, as well as hypocomplementemia (reduced C3 and C4 levels), arthralgia, and cutaneous eruptions.

She received hydroxychloroquine for eight years (200 to 400 mg/day) and occasional courses of systemic glucocorticoids. There were no relevant familial or occupational exposures or passive smoking.

Clinical examination revealed bilateral crackles and urticarial skin lesions. Biological workup showed elevated C-reactive protein (CRP) at 47 mg/L with a normal erythrocyte sedimentation rate (3 mm/h). Autoantibody testing was consistent with previous findings (positive ANA, anti-dsDNA, SSA, SSB) and marked hypocomplementemia (C3 at 0.40 g/L, C4 at 0.05 g/L, CH50 at 59%), without evidence of renal involvement. Alpha-1-antitrypsin levels were normal (Table 1).

**Table 1:** Laboratory findings.

Parameters	Patient Values	Reference Range	Unit
C-reactive protein	47	<5	mg/L
Erythrocyte sedimentation rate	3	0-20	mm/h
Antinuclear antibodies (ANA)	Positive (anti-double-stranded DNA) 1:2560 Identification: SSA, SSB (highly positive)	Positive if ≥ 1:160	
C3 levels	0.40	0.83-1.93	g/L
C4 levels	0.05	0.15-0.57	g/L
CH50	59	>66	%
Creatinin	0.71	0.57-1.1	mg/dL
Alpha-1-antitrypsin	30	20-48	µmol/L

**Figure 1:**
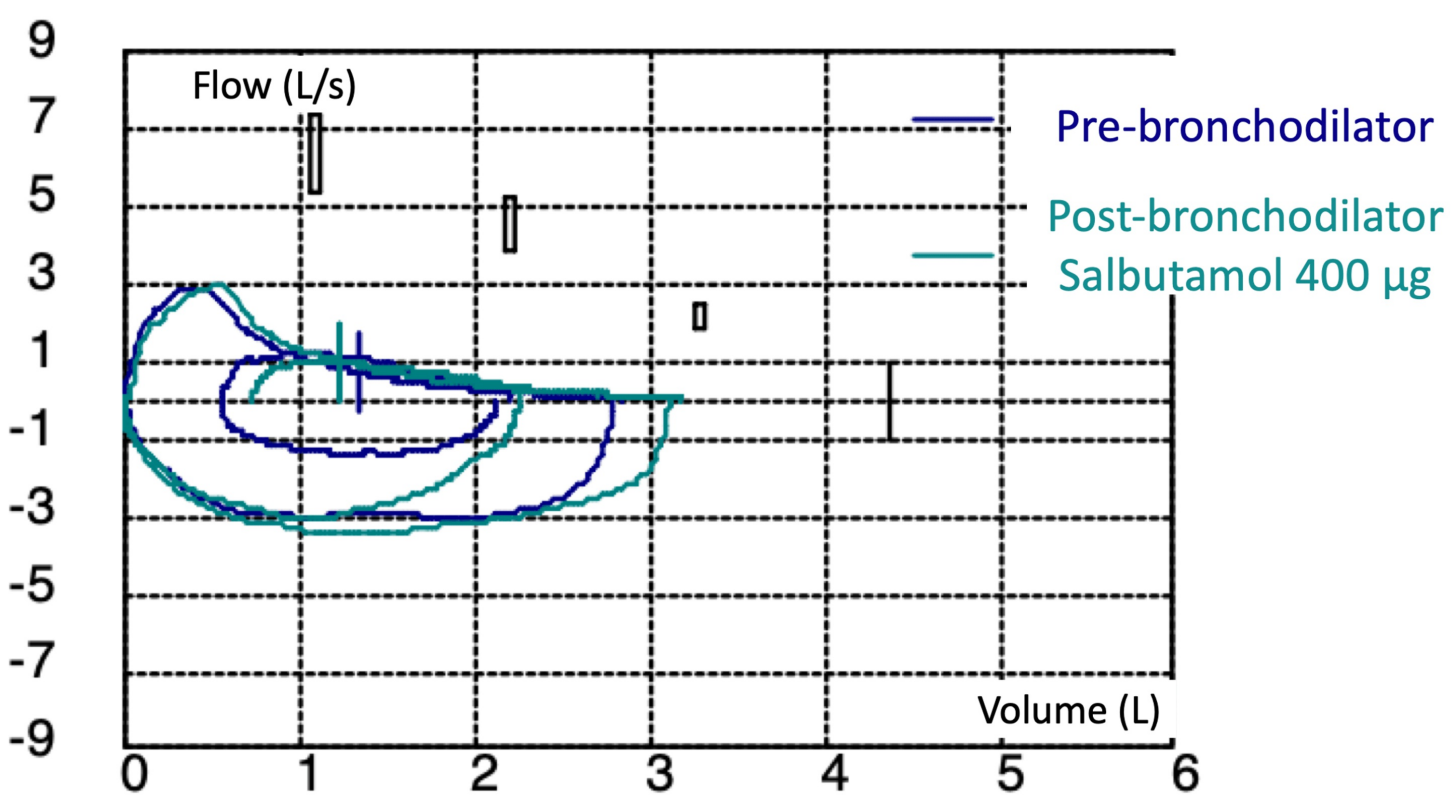
Flow-volume curve in spirometry showing severe obstructive disorder.

Pulmonary function tests indicated a severe obstructive ventilatory defect: forced expiratory volume in one second (FEV₁) at 1.39 L (38% predicted), FEV₁/forced vital capacity (FVC) ratio at 49%, and FVC at 73%, consistent with small airway obstruction, air trapping, and lung hyperinflation (forced expiratory flow (FEF) 25-75% at 21%; total lung capacity (TLC) at 126%, and residual volume (RV) at 256% of predicted values) (Figure 1).

**Figure 2:**
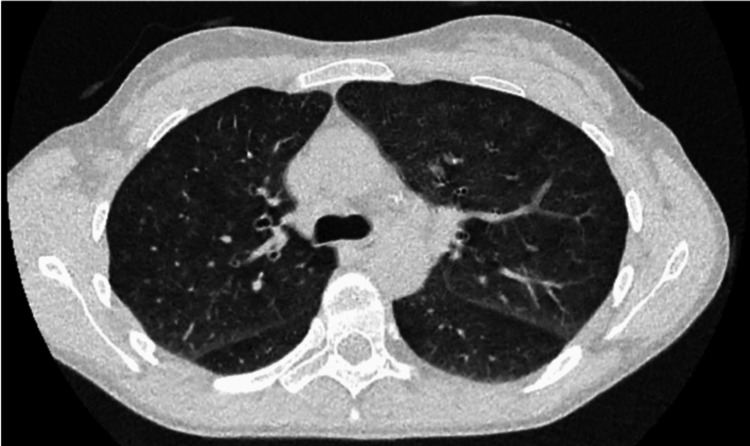
Chest CT scan showing diffuse ground glass opacities.

There was no reversibility with bronchodilator administration. There was also a reduction in alveolar-capillary diffusion (diffusing capacity of carbon monoxide (DLCO) at 46%; transfer coefficient for carbon monoxide (KCO) at 47%). High-resolution chest CT showed interstitial changes with scattered ground-glass opacities suggestive of bronchiolitis, without emphysematous lesions (Figure 2).

Bronchoalveolar lavage (BAL) was unremarkable. The patient declined skin and lung biopsies. Serological tests revealed positive anti-C1q antibodies and undetectable serum C1q levels.

**Figure 3:**
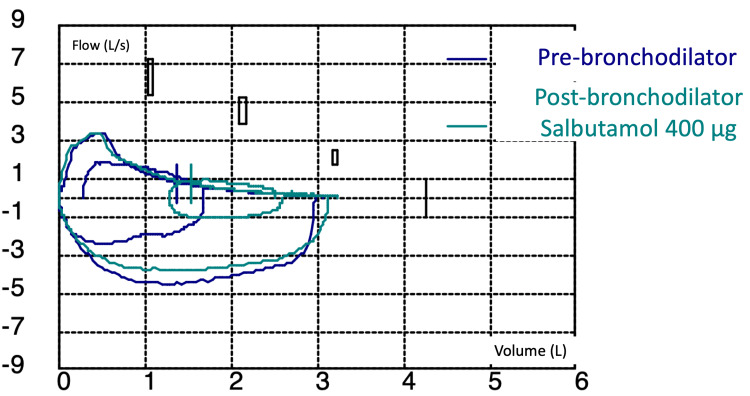
Flow-volume curve in spirometry showing persistent severe obstructive disorder at 12 months of treatment.

Based on this constellation of findings and according to the diagnostic criteria mentioned in the discussion, a diagnosis of bronchiolitis obliterans secondary to HUVS was established. In addition to the previous intermittent corticosteroid regimen, glucocorticoid-sparing treatments were added and included colchicine (at diagnosis), omalizumab, and mycophenolate mofetil (both at 2 months after diagnosis), which quickly led to resolution of extra-pulmonary symptoms and normalization of inflammatory markers. However, during follow-up and at 12 months of therapy, no improvement in pulmonary function was observed. The severe obstructive defect persisted (FEV₁/FVC at 50%, FEV₁ at 41%, FVC at 72%, DLCO at 48%, KCO at 52%) (Figure 3).

**Figure 4:**
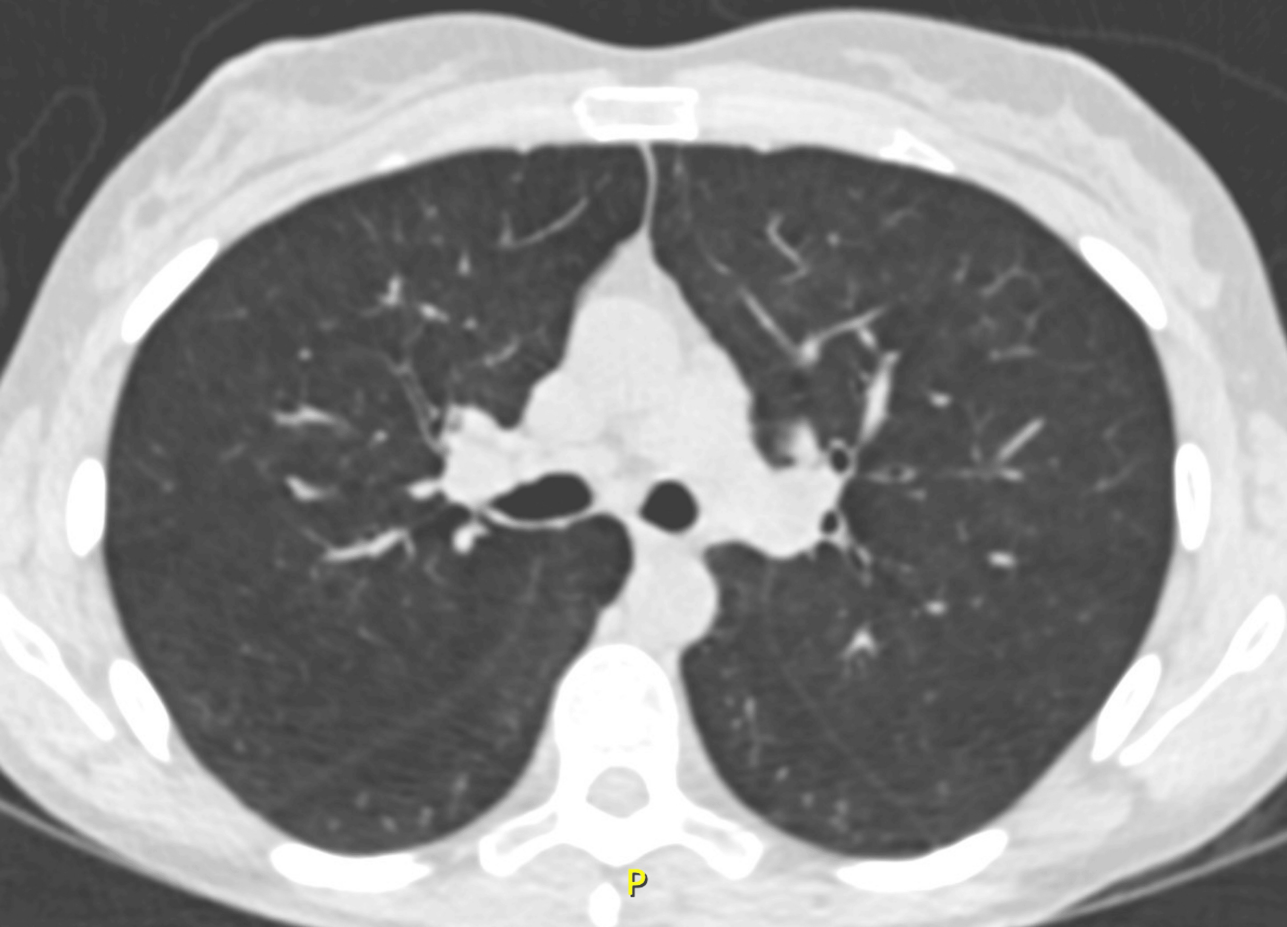
Chest CT scan showing diffuse emphysema.

Follow-up CT imaging showed resolution of acute inflammatory bronchiolitis findings, while demonstrating the development of diffuse pulmonary emphysema (Figure 4), consistent with the development of an irreversible obstructive ventilatory disorder.

## Discussion

HUVS is a rare condition, primarily described through isolated case reports. The presence of urticaria should raise clinical suspicion for this diagnosis, particularly in patients presenting with obstructive ventilatory disorders. The hypocomplementemic form is more severe and has clinico-biological manifestations similar to those of SLE [2,3].

Proposed diagnostic criteria include two major criteria (persistent urticaria for over six months and hypocomplementemia) and several minor criteria shared with SLE (arthritis, glomerulonephritis, uveitis, abdominal pain), as well as more specific findings such as dermal venulitis and positive anti-C1q antibodies with reduced C1q levels [1,3]. Although skin and lung biopsies were declined, the positive anti-C1q antibodies and undetectable C1q levels strongly supported the diagnosis.

Respiratory involvement is typically characterized by obstructive ventilatory impairment, usually associated with a background of smoking-related chronic obstructive pulmonary disease (COPD), with prognosis correlating to cumulative tobacco exposure [1]. In our case, chest CT revealed features consistent with bronchiolitis, including ground-glass opacities and air trapping, in line with the severity of obstruction and elevated residual volume. Recent literature has suggested that tobacco use is strongly associated with severe obstructive disease in affected patients [1,4].

However, our young non-smoking patient initially presented with severe respiratory involvement more consistent with acute bronchiolitis than emphysematous COPD, as previously described. Long-term follow-up showed resolution of biological and imaging inflammatory signs, replaced by severe and diffuse pulmonary emphysema. The literature proposes several potential mechanisms of pulmonary involvement: (1) anti-C1q antibodies targeting collagen-like regions of pulmonary surfactant, (2) pulmonary capillaritis, and (3) alpha-1 antitrypsin dysfunction, potentially explaining emphysema [5]. These mechanisms remain to be fully investigated due to the rarity of the condition and the limited availability of lung biopsy specimens.

Treatment remains challenging, with no guidelines for pulmonary involvement, and is based largely on case reports and small series. Our case underscores the importance of individualizing immunosuppressive therapy, considering agents typically reserved for severe or refractory disease, and raises the question of treatment timing. In this patient, the addition of omalizumab and mycophenolate mofetil to existing therapy led to clinical improvement, particularly in cutaneous and systemic symptoms. The biological inflammatory syndrome and urticaria improved rapidly after treatment escalation, a pattern consistent with previously reported cases [4]. However, at two-year follow-up, no respiratory improvement was observed, with persistent obstructive impairment and unchanged imaging findings, suggesting that lung lesions were already well established at diagnosis and likely irreversible despite intensive combination therapy.

This case highlights the occurrence of severe obstructive lung disease in a young, non-smoking patient with no prior respiratory comorbidities or risk factors for COPD/emphysema, and the irreversible nature of the pulmonary damage despite escalation to quadruple immunosuppressive therapy. The association with urticaria illustrates the relevance of a multidisciplinary approach, as highlighted by a recent article proposing a dermatologic diagnostic algorithm [6]. Implementing such a cross-disciplinary strategy may enhance the early and accurate diagnosis of this rare condition. The pathophysiology remains to be elucidated and reinforces the need for early diagnosis and intervention to prevent irreversible progression.

## Conclusions

The clinical case highlights previously published articles concerning the importance of considering the HUVS diagnosis despite its rarity. Respiratory involvement, which can be severe, dominates the prognosis. Treatment includes various immunosuppressive agents, and there are no recommendations for their use in this entity. Therapies should be guided by the benefit-risk balance according to the severity of the disease.
